# Serum Growth Differentiation Factor 8 (Myostatin) Concentrations in Cats with Early-Stage Chronic Kidney Disease

**DOI:** 10.3390/vetsci13010089

**Published:** 2026-01-15

**Authors:** Kerrigan Fleming, William H. Whitehouse

**Affiliations:** Department of Veterinary Clinical Sciences, College of Veterinary Medicine, Purdue University, West Lafayette, IN 47907, USA; flemin46@purdue.edu

**Keywords:** feline, cachexia, sarcopenia, muscle condition score, weight loss

## Abstract

Weight loss occurs with chronic kidney disease (CKD) and is present in the early stages. Growth differentiation factor 8 (GDF8), also known as myostatin, is a negative regulator of muscle growth, and circulating GDF8 concentrations are increased in people with CKD. The objective of this study was to evaluate if serum GDF8 concentrations in cats with early CKD are increased compared to healthy cats. Associations of GDF8 with age, sex, body weight, body condition score (BCS), muscle condition score (MCS), and selected renal parameters were also examined. Serum GDF8 in healthy (*n* = 10), International Renal Interest Society (IRIS) stage 1 CKD (*n* = 5), and IRIS stage 2 CKD (*n* = 10) cats was quantified using a commercially available sandwich ELISA. GDF8 was not different amongst healthy cats (2137 ± 740 pg/mL) and cats with IRIS stage 1 (1785 ± 530 pg/mL) and IRIS stage 2 (1961 ± 638 pg/mL; *p* = 0.608) CKD. GDF8 was moderately correlated with MCS (*r_s_* = 0.517, 95% CI 0.006–0.814, *p* = 0.049) and inversely correlated with age (*r* = −0.429, 95% CI −0.705 to −0.041, *p* = 0.032), but no association was found with the selected renal parameters, body weight, or BCS.

## Simple Summary

Chronic kidney disease (CKD) is a progressive and prominent disease in cats. Muscle wasting is a common consequence of CKD, with weight loss occurring in the earlier stages of kidney disease. Growth differentiation factor 8 (GDF8) is a protein regulator of muscle growth that promotes muscle breakdown and inhibits muscle synthesis. In people with CKD, GDF8 has been shown to be elevated in serum blood samples. These levels, however, have not been evaluated before in cats with CKD. In this study, GDF8 levels were measured in serum blood samples from healthy cats and cats with International Renal Interest Society stage 1 and stage 2 CKD using a multispecies enzyme-linked immunosorbent assay. GDF8 levels were not different amongst healthy and CKD cats. GDF8 levels were also not different across other markers of kidney function. GDF8 levels decreased with advanced muscle wasting, identifying GDF8 as a potential marker of muscle mass in cats. GDF8 levels also decreased with the age of the cats. With variability in GDF8 production and excretion and potential influence from tissues outside the kidney, further investigation of the role of GDF8 in muscle regulation and cats with CKD is warranted.

## 1. Introduction

Chronic kidney disease (CKD) is a common disease in elderly cats that is permanent and progressive. Loss of functional renal mass is accompanied by a decreased ability to execute normal metabolic and endocrine functions, including clearance of metabolic waste, regulation of acid-base and hydration, and erythropoiesis. Cats with CKD may experience clinical signs such as polydipsia, polyuria, anorexia, muscle loss, and weakness. Cachexia is defined as the loss of muscle associated with underlying illness [1], while the loss of muscle strength that accompanies loss of muscle is the primary characteristic of sarcopenia [2]. Both cachexia and sarcopenia are present with CKD [3], and the loss of muscle mass and function associated with CKD is observed in both people [4] and cats [5]. While increased occurrence and severity of weight and muscle loss is most clinically apparent later in the course of disease, weight loss occurs in the earlier stages of CKD in cats, including International Renal Interest Society (IRIS) stages 1 and 2 [5]. Serving as a negative prognostic indicator, low muscle mass and/or body weight is associated with worse outcomes in both human and veterinary CKD populations [5,6,7,8,9].

Protein synthesis is regulated by two pathways: the insulin-like growth factor-1/Akt pathway promoting protein synthesis and the myostatin pathway promoting proteolysis [4]. Myostatin, also known as growth differentiation factor 8 (GDF8), is a negative regulator of protein synthesis and thus muscle growth, playing a role in the pathogenesis of both sarcopenia and cachexia. The original discovery of GDF8 was when skeletal muscle hyperplasia and hypertrophy were found to result from GDF8 gene deletion [10]. Similar results are reported in other species [11,12,13]. Consequently, its main physiological role is to prevent muscle hypertrophy, and the desirable traits of loss-of-function genetic mutations of GDF8 have sparked research and influenced breeding programs in the meat production industry [14]. In cats, decreased circulating GDF8 concentrations might play a role in the development of congestive heart failure from hypertrophic cardiomyopathy [15]. Unfortunately, knowledge of its function in other disease states in this species is significantly lacking.

Little is known about gain-of-function genetic mutations of GDF8, but variants in the human GDF8 gene are thought to play a role in the development of sarcopenia [16]. Nevertheless, increased GDF8 gene expression is a well-documented and important cause of skeletal muscle atrophy, which can result from a variety of acquired factors [17,18,19]. These findings have led to the investigation of GDF8 inhibitors as a therapeutic for muscle wasting associated with many disorders [20,21,22,23,24].

GDF8 is a member of the transforming growth factor-β superfamily that is primarily secreted by skeletal myocytes [10]. GDF8 favors muscle breakdown through activation of the ubiquitin-proteasome pathway, inhibition of satellite muscle cell recruitment, and inhibition of protein synthesis through suppression of the anabolic Akt/mechanistic target of rapamycin signaling pathway [4,25,26]. With an increase in inflammatory cytokines, oxidative stress, and decreased muscle activity, CKD is a likely proponent for increased circulating GDF8 concentrations [4,26]. In people, blood GDF8 concentrations were found to be increased in CKD [27,28,29], including the early stages [28,29]. While there is evidence for increased GDF8 concentrations in people with early-stage CKD, GDF8 concentrations in cats with CKD have not been examined. The primary objective of this study was to evaluate if serum GDF8 concentrations in IRIS stage 1 and 2 CKD are increased compared to healthy cats without CKD. Secondary objectives were to investigate the association of GDF8 concentrations with selected population and renal function parameters. Our hypothesis was that serum GDF8 concentrations would be higher in cats with CKD compared to healthy cats without CKD. We also hypothesized that there would be a positive correlation with serum GDF8 and markers of a decline in renal function.

## 2. Materials and Methods

### 2.1. Study Cohort and Sampling

A retrospective case–control study design was utilized using samples banked from a previous study of prospectively enrolled cats with CKD as well as healthy cats ≥ 7 years of age without CKD. Client-owned cats were enrolled after informed consent was obtained at Kansas State University Veterinary Health Center between March and November 2022. CKD was staged according to IRIS guidelines based on serum creatinine, symmetric dimethylarginine (SDMA), and urine specific gravity values from clinically stable patients on 2 separate visits approximately 3 weeks apart [30]. All cats were fasted for 10–12 h prior to the appointment. Concurrent disease was ruled out in all cats based on history, physical examination, complete blood count, biochemistry panel, urinalysis, total thyroxine level, abdominal radiographs, and abdominal ultrasound performed by a board-certified radiologist or resident under the direct supervision of a board-certified radiologist. Body condition score (BCS) and muscle condition score (MCS) were recorded for all cats by a single investigator (WW) in accordance with the World Small Animal Veterinary Association Global Nutrition Committee guidelines [31,32]. Serum samples from these cats were collected at the second baseline visit and stored at −80 °C until analysis. Selected population parameters included age, sex, body weight, BCS, and MCS, and renal function parameters included serum creatinine, blood urea nitrogen, SDMA, phosphorus, and urine specific gravity (USG). Additionally, serum potassium and creatine kinase (CK) were assessed.

### 2.2. ELISA Procedure

Serum GDF8 concentrations were measured using a commercially available multispecies quantitative sandwich enzyme-linked immunosorbent assay (GDF-8/Myostatin; DGDF80; R&D Systems, Inc., Minneapolis, MN, USA) as the homology of GDF8 is relatively conserved, with feline GDF8 sharing 97% amino acid sequence identity with canine GDF8 and 92% with human GDF8 [33]. The assay procedure was performed according to the manufacturer’s specifications and has been previously described in detail [34]. The final dilution factor of the serum samples was 1:50, and samples were examined in triplicate. The calibration curve was made with concentrations ranging from 0 pg/mL to 2000 pg/mL. A microplate reader (Synergy™ HT, Biotek^®^ Instruments, Inc., Winooski, VT, USA) was used to determine the optical density of each well, and final concentrations were determined from the difference in the readings at 540 nm from 450 nm to correct for optical imperfections in the plate.

### 2.3. Statistical Analysis

Sample size calculation was performed using commercially available software (G*Power 3.1.9.7, Düsseldorf, Germany). Based on GDF8 concentrations in a cohort of healthy cats ≥ 6 years of age, 5 cats in each group would provide 80% power to detect, as significant, a 30% difference in GDF8 [15]. Continuous variables were assessed for normality with the Shapiro–Wilk test. Normally distributed data are presented as mean ± standard deviation, and non-normally distributed data are presented as median (range). For analysis, MCS categories were given the following numerical values: normal muscle mass = 3, mild muscle loss = 2, moderate muscle loss = 1, severe muscle loss = 0. Comparisons amongst the healthy cats, cats with IRIS CKD stage 1, and cats with IRIS CKD stage 2 were made using one-way analysis of variance or the Kruskall-Wallis test as appropriate, followed by Tukey’s or Dunn’s post hoc test. Evaluations between two groups were performed using an unpaired *t*-test or Mann–Whitney U test. Correlations with GDF8 concentrations were assessed with Pearson’s or Spearman’s rank correlation coefficient. Correlation based on the *r* or *r*_s_ coefficient was interpreted as weak (0.1–0.39), moderate (0.4–0.69), strong (0.7–0.89), or very strong (0.9–1.0) [35]. Statistical significance was set at *p* < 0.05. Statistical analysis was performed using Prism 10.4.2, GraphPad Software, Inc., San Diego, CA, USA.

## 3. Results

A total of 32 cats were enrolled. Three were excluded because of suspected neoplasia identified on abdominal ultrasound (*n* = 1 urinary bladder mass, *n* = 1 hepatic mass, *n* = 1 cecal mass). Cats with uncontrolled hyperthyroidism (*n* = 1), IRIS CKD stage 3 (*n* = 1), suspected acute kidney injury (*n* = 1), and dilute urine suspected to be from eating a diet formulated for the prevention of uroliths (*n* = 1) were also excluded. Twenty-five cats remained in the study and were categorized as IRIS CKD stage 1 (*n* = 5), IRIS CKD stage 2 (*n* = 10), and healthy cats without CKD (*n* = 10).

**Figure 1:**
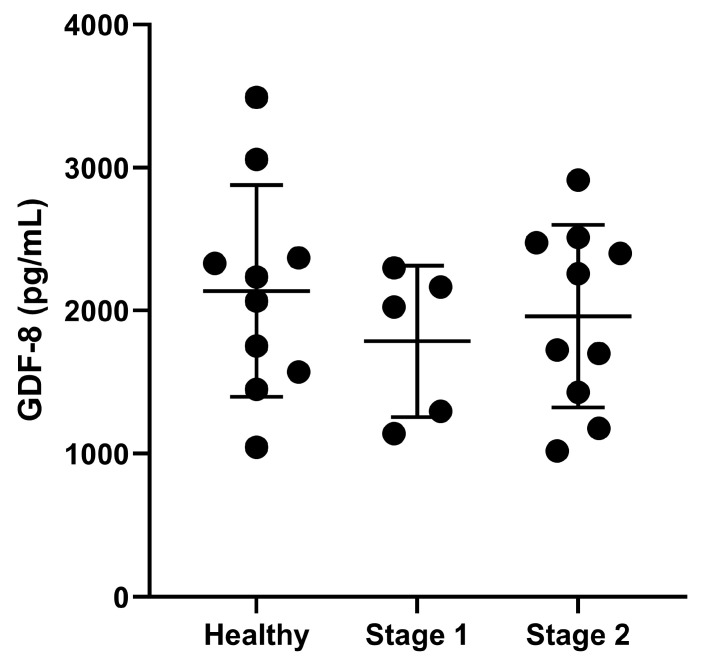
Serum growth differentiation factor 8 (GDF8) concentrations in pg/mL in healthy cats (*n* = 10) and cats with International Renal Interest Society chronic kidney disease stage 1 (*n* = 5) and stage 2 (*n* = 10). Serum GDF8 was not significantly different across the study groups (*p* = 0.608). The dots represent individual data points, and the bars represent mean and standard deviation.

All of the continuous variables met the normality assumption except for CK. Study population demographics evaluated, including age, sex, breed, body weight, BCS, and MCS, along with the selected renal function parameters, are summarized in Table 1. The intra-assay coefficient of variability was 2.88%. Serum GDF8 concentrations were not different amongst the healthy (2137 ± 740 pg/mL), IRIS CKD stage 1 (1785 ± 530 pg/mL), and IRIS CKD stage 2 cats (1961 ± 638 pg/mL, *p* = 0.608; Figure 1). There was no association found between GDF8 concentrations and any of the selected markers of renal function.

**Figure 2:**
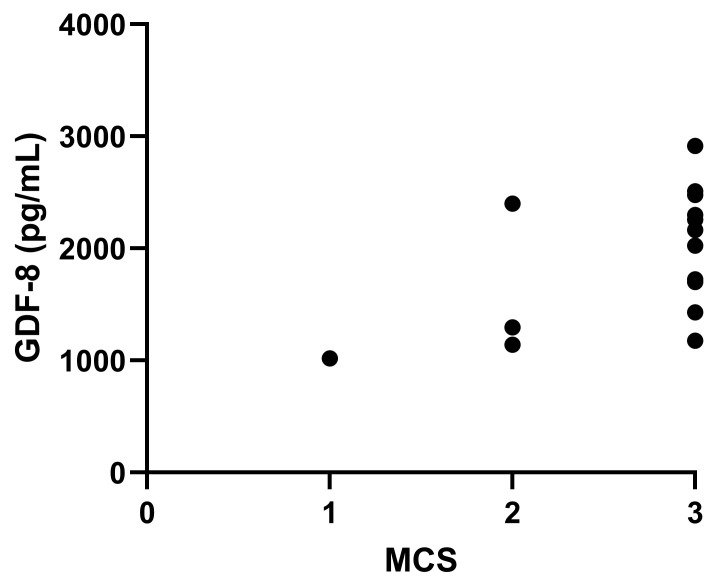
Moderate correlation between serum growth differentiation factor 8 (GDF8) concentrations in pg/mL and muscle condition score (MCS) in cats with chronic kidney disease (*r_s_* = 0.517, *p* = 0.049). The dots represent individual data points.

**Figure 3:**
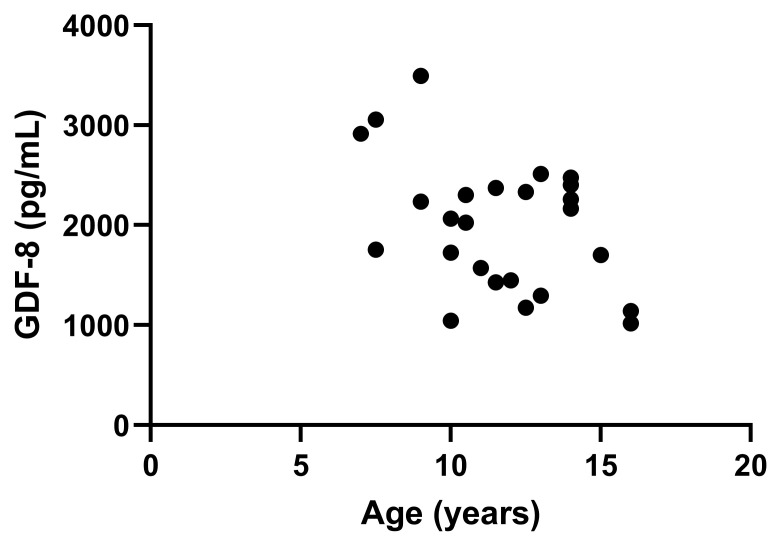
Moderate inverse correlation between serum growth differentiation factor 8 (GDF8) concentrations in pg/mL and age in years in cats (*r* = −0.429, *p* = 0.032). The dots represent individual data points.

**Figure 4:**
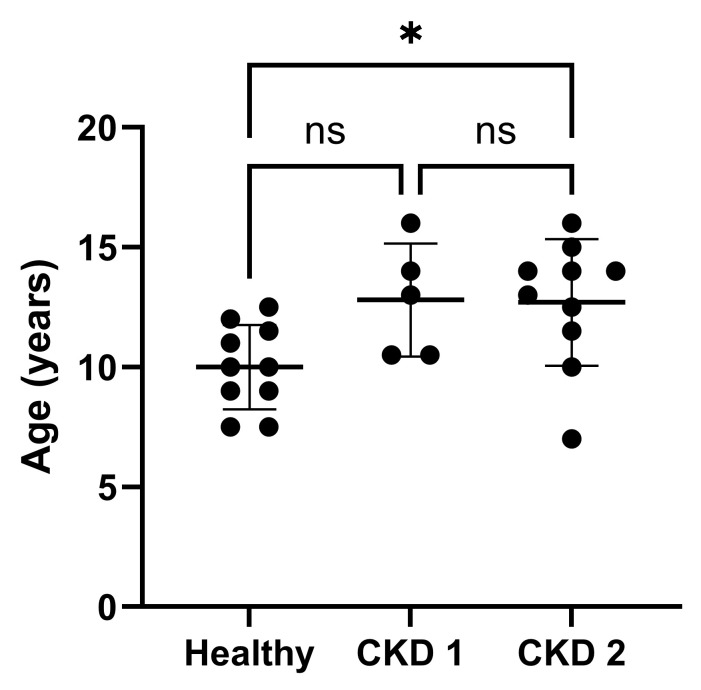
Age of healthy cats and cats with International Renal Interest Society (IRIS) chronic kidney disease (CKD) stage 1 and 2. Cats with IRIS CKD stage 2 were significantly older than healthy cats (*p* = 0.036). The dots represent individual data points, and the bars represent mean and standard deviation. * = statistically significant with *p* < 0.05, ns = not significant.

Serum GDF8 of cats with CKD was moderately correlated with MCS (*r*_s_ = 0.517, 95% CI 0.006–0.814, *p* = 0.049; Figure 2). GDF8 was also negatively correlated with age (*r* = −0.429, 95% CI −0.705 to −0.041, *p* = 0.032; Figure 3). Age of the cats was found to be significantly different across the IRIS CKD stages and healthy cats (*p* = 0.025; Figure 4), with the IRIS CKD stage 2 cats (12.7 ± 2.64 years) being significantly older than the healthy cats (10 ± 1.76 years, mean difference 2.7, 95% CI 0.16–5.25, *p* = 0.036). Additionally, there was a moderate negative correlation between MCS and age (*r*_s_ = −0.497, 95% CI −0.751 to −0.0114, *p* = 0.012), but MCS was not significantly different across the IRIS CKD stages and healthy cats (*p* = 0.708). There was no association found between GDF8 concentrations and body weight or BCS.

No cat was considered hypokalemic based on the laboratory’s reference interval (3.4–4.9 mmol/L), and there was no difference in potassium amongst the study groups (*p* = 0.123). Additionally, potassium did not correlate with GDF8 (*p* = 0.268) or MCS (*p* = 0.538). Serum CK activity was also not different amongst the study groups (*p* = 0.426), and no correlation was found between CK and GDF8 (*p* = 0.924), MCS (*p* = 0.174), or potassium (*p* = 0.938).

## 4. Discussion

In this study, serum GDF8 concentrations were not significantly different in cats with IRIS CKD stage 1 or 2 compared to healthy cats without CKD. Additionally, there was a moderate positive correlation between GDF8 and MCS as well as a moderate inverse correlation between GDF8 and age. Production of GDF8 is stimulated by a variety of factors. Uremic toxins, oxidative stress, inflammation, metabolic acidosis, angiotensin II, and physical inactivity all lead to increased circulating concentrations and/or mRNA expression of GDF8 [17,36,37,38,39]. Consequently, CKD provides an opportune environment for GDF8 upregulation. GDF8 is also thought to accumulate secondary to decreased renal clearance [28,40].

Although most studies evaluating circulating concentrations of GDF8 in people with CKD show an increase compared to healthy controls [28,29,41,42], some studies found no difference in GDF8 concentrations between CKD patients and controls [43,44,45]. The main source of GDF8 production is skeletal myocytes [10]. Within skeletal muscle, GDF8 acts as a chalone protein primarily in a paracrine and autocrine manner to locally regulate muscle growth [46]. Similarly to this study, there are several investigations in human CKD patients that found a positive association between serum GDF8 concentrations and muscle mass [43,47,48]. Additionally, there is variability in muscle mRNA expression of GDF8 with CKD in people and rodents, with studies showing increased [37,49,50] and decreased expression [40]. Consequently, muscle mass appears to influence circulation concentrations of GDF8, and circulating GDF8 concentrations may not be an accurate representation of muscle GDF8 activity. Furthermore, GDF8 is produced from other cell types such as adipose and cardiac tissue [10,51]. A significant association between GDF8 and BCS was not found in this study. Unfortunately, none of the cats had a diagnostic evaluation performed to rule out cardiac disease. Only 6 cats had a heart murmur heard on auscultation: healthy (*n* = 3), IRIS CKD stage 1 (*n* = 1), and IRIS CKD stage 2 (*n* = 2); however, the absence of a heart murmur does not exclude the presence of an underlying cardiomyopathy [52]. The complex interplay between GDF8 production and excretion with CKD, as well as the potential influence from extra-renal tissues, could result in the variability in circulating GDF8 concentrations across studies.

A moderate inverse correlation between serum GDF8 concentrations and age was identified in the cats in this study. This is similar to findings in people [45,47] and dogs [34,53]. Declining GDF8 concentrations with age could be a compensatory physiologic mechanism when sarcopenia is present, but further studies are needed to confirm this notion [25]. In this study, there was also a moderate inverse correlation with MCS and age; therefore, the association found between GDF8 and age could be more of a reflection of the muscle mass present and not age itself.

To the authors’ knowledge, there is one other study investigating circulating GDF8 concentrations in cats [15]. In that study, cats with hypertrophic cardiomyopathy and a history of congestive heart failure had lower GDF8 concentrations compared to cats without cardiac disease and cats with hypertrophic cardiomyopathy or hypertrophic obstructive cardiomyopathy that did not have a history of congestive heart failure. It is speculated that the lower circulating levels could represent a decrease in cardiomyocyte numbers due to an increase in cardiac fibrosis, but cardiac biopsies would be needed to confirm this hypothesis. There were no differences with GDF8 in relation to body weight or BCS in those cats, findings consistent with this study. No difference in GDF8 concentrations based on age was found, but that could be a result of similar MCS amongst the study groups. MCS was not being assessed in that study [15]. It is difficult to render many comparisons between these two studies given the different disease states evaluated; however, the decreased circulating concentrations of GDF8 found in both studies might signify that it has the ability to indicate muscle wasting in cats. The lack of data on GDF8 in cats highlights the need for further inquiry into its role in pathophysiology and effect on outcomes in this species. In people with CKD, GDF8 promotes tubulointerstitial inflammation [54] and is associated with endothelial dysfunction [55].

Although there does not appear to be a direct relationship between GDF8 and potassium, hypokalemia is a relatively common finding in cats with CKD [56] and a known cause of muscle dysfunction [57]. Consequently, potassium was evaluated as another variable in this study. None of the cats were hypokalemic; however, 2 cats in each group did have values < 4 mmol/L. Though normokalemic cats may still have decreased muscle potassium content [58], no association was found between potassium concentrations and CK activity. Additionally, we did not identify any associations between these variables and MCS or GDF8. These results may be a reflection of the small sample size and selection of very stable cats with CKD.

There are several important limitations of this study to consider. Only cats with CKD that were classified as IRIS stage 1 or 2 were included in this study, which precluded cats with more advanced renal disease and likely more profound muscle wasting. Since one of the main clinical goals of CKD management is to slow the progression of disease, we were particularly interested in evaluating GDF8 in cats with early-stage CKD since weight loss is already apparent at this point [5]. However, it is unclear how circulating GDF8 concentrations change in the more advanced stages of CKD in cats. This study utilized samples banked from a previous prospective study of cats with well-characterized early CKD. Although a standardized diagnostic evaluation was performed with each cat to rule out concurrent disease, the possibility for comorbidities remains, particularly cardiac disease, since echocardiography was not performed. Another limitation of this study includes the lack of assessment of muscle strength. In one study, serum GDF8 concentrations were not associated with skeletal muscle index but were associated with sarcopenia dictated by hand grip strength in human CKD patients [59]. The decline in muscle function with CKD is an important contributor to the decrease in quality of life with these patients, which warrants further exploration of both causes and potential therapeutic targets. Correlation of GDF8 to other variables affecting its expression, such as other uremic toxins, markers of inflammation and oxidative stress, and angiotensin II levels, was also not performed in this study. The sample size in this study was also small and likely resulted in the wide confidence intervals.

## 5. Conclusions

In this study, serum GDF8 concentrations were not different among IRIS CKD stage 1 cats, IRIS CKD stage 2 cats, and healthy cats without CKD. There was also a moderate positive correlation between GDF8 and MCS. The inverse correlation between GDF8 and age in these cats is thought to be related to the decrease in muscle mass with age. This study highlights the potential for circulating GDF8 to be a biomarker reflecting muscle wasting in cats. Further studies are needed to better define its role in aging and CKD in cats.

## Figures and Tables

**Table 1:** Select demographics and renal parameters of healthy cats without chronic kidney disease (*n* = 10) and cat with International Renal Interest Society chronic kidney disease stage 1 (*n* = 5) and stage 2 (*n* = 10).

	Healthy	CKD Stage 1	CKD Stage 2	*p*-Value
Age (years)	12.7 ± 2.64	12.8 ± 2.36	10 ± 1.76	0.025
Sex	Female (*n* = 2) Male (*n* = 8)	Female (*n* = 2) Male (*n* = 3)	Female (*n* = 4) Male (*n* = 6)	
Breed	DLH (*n* = 1) DSH (*n* = 9)	DLH (*n* = 2) DMH (*n* = 1) DSH (*n* = 1) Manx (*n* = 1)	Bengal (*n* = 1) DMH (*n* = 3) DSH (*n* = 6)	
Weight (kg)	4.6 ± 1.09	4.7 ± 1.01	5.2 ± 1.38	0.540
BCS (out of 9)	5.5 (4–8)	4 (3–7)	7 (2–8)	0.228
MCS (out of 3)	3 (2–3)	3 (2–3)	3 (1–3)	0.708
Creatinine (mg/dL)	1.27 ± 0.22	1.2 ± 0.19	2 ± 0.31	<0.001
BUN (mg/dL)	24.1 ± 4.2	25.8 ± 4.15	32.7 ± 7.33	0.008
SDMA (µg/dL)	9.3 ± 2.21	13.4 ± 2.7	15.8 ± 2.9	<0.001
Phosphorus (mg/dL)	3.88 ± 0.71	3.88 ± 0.52	4.59 ± 0.73	0.06
USG	1.047 ± 0.007	1.029 ± 0.01	1.025 ± 0.01	<0.001
Potassium (mmol/L)	4.16 ± 0.28	3.98 ± 0.39	4.45 ± 0.55	0.123
CK (U/L)	161.5 (96–590)	187 (68–369)	213 (132–876)	0.426

Abbreviations: BCS = body condition score, BUN = blood urea nitrogen, CK = creatine kinase, CKD = chronic kidney disease, DLH = domestic longhair, DMH = domestic mediumhair, DSH = domestic shorthair, MCS = muscle condition score, SDMA = symmetric dimethylarginine, USG = urine specific gravity.

## Data Availability

The data presented in this study are available on request from the corresponding author. The raw data are not publicly available due to patient/client privacy restrictions.
